# Study protocol: Effectiveness of patient centered pharmacist care in improving medication adherence, clinical parameters and quality of life among hemodialysis patients

**DOI:** 10.1371/journal.pone.0263412

**Published:** 2022-02-18

**Authors:** Ganesh Sritheran Paneerselvam, Raja Ahsan Aftab, Roland Gamini Sirisinghe, Pauline Siew Mei Lai, Soo Kun Lim

**Affiliations:** 1 School Of Pharmacy, Faculty of Health and Medical Science, Taylor’s University, Selangor, Malaysia; 2 Department of Primary Care Medicine, Faculty of Medicine, University of Malaya, Kuala Lumpur, Malaysia; 3 Department of Medicine, Faculty of Medicine, University of Malaya, Kuala Lumpur, Malaysia; University of South Australia, AUSTRALIA

## Abstract

**Background:**

Patients requiring hemodialysis (HD) often have several chronic comorbidities, which necessitate the use of several medications and hence put them at high risk of polypharmacy. Medication-related problems (MRPs) among HD patients are a serious issue as they can increase morbidity and nonadherence with medications. To overcome this issue, a unique pharmacy practice model including medication review (MR) and motivational interviewing (MI) is needed to improve medication adherence, by reducing MRPs and optimizing therapeutic outcomes. The present study aims to assess the effectiveness of MR and MI in improving medication adherence, quality of life (QOL) and clinical outcomes among end-stage renal disease (ESRD) patients who are on dialysis.

**Method and design:**

This pre-post study will be conducted prospectively among patients with ESRD who have been on dialysis at the Hemodialysis Unit, Hospital Kuala Lumpur and the Hemodialysis Affiliated Centers of the University Malaya Medical Centre, from August 2020 till August 2021. Medication adherence will be assessed using the General Medication Adherence Scale (GMAS), whilst patients’ HRQOL will be assessed using the Kidney Disease Quality of Life Short Form 36 (KDQOL-36). Clinical parameters such as blood glucose level, calcium, phosphate, hemoglobin and serum low-density lipoprotein (LDL) levels will be obtained from medical records. A total of 70 patients will be recruited.

**Discussion:**

We hypothesize that the implementation of pharmacy-based MR and MI may expect an increase in medication adherence scores and increase in HRQOL scores from baseline as well as achieving the clinical lab parameters within the desired range. This would indicate a need for a pharmacist to be involved in the multidisciplinary team to achieve a positive impact on medication adherence among hemodialysis patients.

**Trial registration:**

Ethical approval has been obtained from the National Medical Research and Ethics Committee NMRR: 20-1135-54435 and Medical Research Ethics Committee, University Malaya Medical Centre MREC ID NO: 202127-9811.

## Introduction

The Global Burden of Disease study (2010) ranked chronic kidney disease (CKD) as the 27th highest cause of total global deaths in 1990, and the 18th highest in 2010 [1]. In Malaysia, a total of 31,497 of patients went for HD in 2014, compared to 29,192 patients in 2013. According to the Malaysian Dialysis and Transplant Registry 2018, the number of new dialysis patients has increased yearly with 8431 new cases reported (an increase of 5%) in 2018. An improvement in the survival rates of patients with ESRD can be seen, due to advances in dialysis treatment [2]. However, the mortality rates for patients with ESRD on chronic dialysis remain high [3] due to the presence of inflammation, undernutrition, and heart-related problems [4, 5].

In addition, ESRD patients who are on dialysis show an impaired quality of life [6]. This is because these patients are not able to accept their disease state, since undergoing renal replacement therapy may make them feel stigmatized or pressured as they are not as "normal" as their friends [7, 8] besides the extensive time spent in dialysis, high expenses and lifestyle restriction to diet and fluid intake, also becomes the contributing factors of low quality of life among HD patients [9]. Generally, the health-related quality of life (HRQOL) refers to the physical, psychological, and social functioning of a person [10]. It is a significant marker of how the ESRD patients are coping with their disease [11]. Assessing health-related quality of life can show the degree of burdens in the ESRD patients [2]. A cross-sectional study showed that ESRD patients who are on dialysis reported to have highly impaired HRQOL, both physically and mentally [12]. Thus, it is vital to identify the potential determinants for lower quality of life to ensure appropriate intervention is being made.

Patients requiring HD often have several chronic comorbidities, which necessitate the use of several medications and hence put them at high risk of polypharmacy. Subsequently, the prevalence of polypharmacy (use of 5 or more medications) among patients requiring HD is high, which leads to medication-related problems, especially in ESRD patients who are on dialysis. It is estimated that at least one MRP is identified for every 15.2 drug exposures [13]. MRPs among ESRD patients who are on dialysis are a serious issue as they can induce morbidity, and nonadherence to prescribed oral medications. Poor compliance and nonadherence are a public health issue in which a patient’s beliefs and myths such as uncertainty on the efficacy of the treatment; nonacceptance of the disease; disbelief in the diagnosis plays a role in shaping their attitude and practice toward medications. Moreover, lower socioeconomic status and educational level are the contributing factors to nonadherence as well [14]. Studies shown that an average prevalence rate of 52.5% medication non- adherence was reported among HD patients [15].

Thus, adherence to medication therapy is a key component of the effective management of dialysis patients. The pharmacist plays a crucial role in identifying MRPs associated with polypharmacy and suggests appropriate interventions to optimize patient outcomes. Moreover, including pharmacists in multidisciplinary team rounds, pharmacist activity such as medication therapy management, medication review and reconciliation as well as deprescribing [16] have proven to improve patient’s adherence to their medications and dietary regimens by minimizing polypharmacy [17]. However, for interventions that are complex and require lifestyle modifications it is worth addressing patients’ beliefs and intentions to perform action. A more comprehensive approach entails a full review of drug regimens and patient attitudes are needed rather than patient education or counseling alone [18].

Therefore, recommendations involving complex behavior change through MI are required. Motivational interviewing is a skillful clinical method, a style of counseling and psychotherapy that is widely used in medical settings to promote autonomy for self-direction based on patient goals and values [19]. A pharmacy-based MI and review program have an important impact on the care of hemodialysis patients by reducing the number of MRPs.

Information about the quality of life and adherence among hemodialysis patients in Malaysia is limited to reporting the incidence rate. To date, there is a paucity of information regarding the effectiveness of reducing MRPs and improving adherence using MI techniques among ESRD patients who are on dialysis [20]. In addition, there is no MI component in the renal medication therapy adherence clinic practice. The relationship between MRPs and QOL among HD patients has rarely been studied in Malaysia. Moreover, a previous study on improving adherence among HD patients uses only one component, either MR or MI, to show effectiveness. To date, no study has used a combination of these two interventions to create sustainable behavioral change for adherence to medication. **Hence, this study will evaluate the effectiveness of MR and MI in improving dialysis patients’ adherence, QOL and clinical outcomes.**

## Materials and methods

### Study design

A pre-post study will be conducted prospectively among hemodialysis patients at the Hemodialysis Unit, Hospital Kuala Lumpur (HKL), and the Hemodialysis Affiliated Centers of the University Malaya Medical Centre (UMMC). The selected patients will be followed for a period of 12 months. The estimated sample size using a power of 0.95 and 95% confidence interval was 56 patients.


$$n>={\left\{\begin{array}{c} Z_{\alpha /2}+Z\beta \\ ES \end{array}\right\}}^{2}$$

n = sample size

Z = statistic for level of confidence, using a 95% confidence interval (so Z = 1.96)

α = 0.05

β = type 11 error

ES = Effect size, δ / σ

δ = A difference in population means

σ = Standard deviation of difference in the response of matched pairs

Therefore, by using value of δ = 0.9 and σ = 1.84 from similar previous study on chronic illness patient [21].

n = 56

Study of a continuous response variable from matched pairs of study subjects. Prior data indicate that the difference in the response of matched pairs is normally distributed with standard deviation 1.84. If the true difference in the mean response of matched pairs is 0.9, we will need to study 56 pairs of subjects to be able to reject the null hypothesis that this response difference is zero with probability (power) 0.95. The Type I error probability associated with this test of this null hypothesis is 0.05.

**≈** 56 ± 25% * (to compensate for drop off or incomplete data)

≈ 70 respondents

*considering dropout rate of 25%, 14 patients were added; therefore total will be 70 patients altogether.

#### Participants

All patients aged 18 years or over undergoing hemodialysis treatment (thrice a week) for at least 3 months, and able to communicate in English or Malay, will be included in the study. Patients who have had any major surgical interventions in the previous three months, or have malignancies, cognitive impairment, dementia, active psychosis, or major hearing impairment, or are pregnant or breastfeeding, will be excluded.

#### Patient selection

A simple random sampling technique with a research randomizer, an online tool, will be used to recruit potential patients [22]. The list of all hemodialysis patients will be entered into the research randomizer to generate random numbers to assign to patients for selection [23]. This method of computer-generated simple randomization can minimize the risk of selection bias, as patients will be selected by chance [24]. All demographic data of the selected patients such as age, gender, past medical & medication history, dialysis history, medications prescribed (name, dose, frequency, route, duration of the drug) will be obtained from electronic health records. Once all potential patients are selected, a written, informed consent will be taken from the patients prior to participation.

#### Patient centered pharmacist care

This study will implement a combination of two methods of patient centered pharmacist care, medication review and motivational interviewing. MR helps in identifying, resolving, and preventing drug related problems. While MI is a collaborative communication approach between the pharmacist and patient to stimulate motivation to improve medication adherence.

### Outcomes measured

#### Primary outcome

Measuring the number of medication-related problems. All medication-related problems will be classified using the Pharmaceutical Care Network Europe (PCNE) classification version 9.00, which was last updated in June 2019 [25]. This is an established system for medication-related problem classification. With it, medication-related problems are categorized into nine primary domains for DRP causes. This tool has been validated to measure medication related problems among chronically ill patients [26]. Medication discrepancies are categorized in Table 1. Identification of medication record discrepancies included in this study follows previous literature [27, 28].

**Table 1:** Medication record discrepancy.

Unintentional Discrepancy: medication change made either inadvertently or deliberately by the patient without the knowledge of the health care team. Undocumented Intentional Discrepancy: medication change made by another healthcare professional but not listed on the medication record. Subcategories include: ● omission (medications being taken by the patient but not listed in the medication record) ● commission (medications no longer being taken by the patient but still listed on the medication record) ● wrong drug ● wrong dose ● wrong frequency ● dose/schedule not listed

Source: Medication record discrepancy [22,23].

#### Secondary outcomes

Measuring the number of adherences, quality of life and clinical outcomes.

*a) General Medication Adherence Scale (GMAS)*. A novel GMAS will be used to measure the adherence. This scale consists of 11 questions which measure the adherence in three domains: the first 5 questions measure nonadherence due to patient behavior, followed by 4 questions measuring the comorbidity and pill burden related nonadherence, and the final 2 questions measuring cost-related nonadherence. Each question has 4 possible answers—always, mostly, sometimes, and never—which will be awarded a score of 0, 1, 2 or 3, respectively. A cumulative medication adherence score of 30 to 33 will be considered as high adherence, followed by a score of 27 to 29 as good adherence, a score of 17 to 26 as partial adherence, a score of 11 to 16 as low adherence and lastly poor adherence for patients scoring 0 to 10. This adherence tool has been validated to measure medication adherence among chronically ill patients [29].

*b) Kidney disease quality of life-36*. A validated KDQOL-36 questionnaire will be used to measure the quality of life among HD patients. KDQOL-36 is a disease-specific instrument that has been widely used to assess the HRQOL in ESRD patients. It contains a subset of the KDQOL-SF items, including the SF-12 items and 24 items to obtain three kidney disease-specific scales, which are burden of kidney disease (4 items), effects of kidney disease on daily life (8 items), and symptoms or problems (12 items). The three disease-specific subscales can be summated into the kidney disease component summary score. The SF-12 is developed using a subset of the SF-36 items. It will generate two summary scores, which are the physical component summary (PCS) and mental component summary (MCS). All these scores range from 0 to 100, with higher scores reflecting better health. In this study, the Malay version of the KDQOL-36 questionnaire will be used, as it is validated [30].

*c) Clinical outcomes*. Clinical outcomes such as blood glucose level, calcium, phosphate, hemoglobin and LDL levels will be obtained from the computer and medical records at the HD unit. These lab parameters will be measured at the baseline, 6^th^ month and the final of the study. The changes in the value of the clinical parameters will be analyzed to find if there is any significant difference after the intervention.

This analysis is important as it acts as surrogate marker for adherence and can be corelated with GMAS scores to show that lab parameters will be within the desired range if patient adherence is improved.

### Interventions

Two interventions will be provided in this study: medication review and motivational interviewing.

#### a) Medication review

MR is a well-structured method to optimize medication use and improve health outcomes among patients [31]. The researcher will examine patient medical records and interview patients to identify patient medication discrepancies and medication-related problems. Any medication issue related to a patient’s medicine-taking behavior and use of medicine in the context of their clinical condition will be recorded. The PCNE version 9 and kidney-related clinical practice guidelines will be used as guides. All the recorded medication problems will be discussed with relevant physician. The changes done by the physician will be notified to the researcher and can be tracked in the following follow up.

#### b) Motivational interviewing

Motivational interviewing is an interactive counseling style in which patients are engaged in the process of thinking and talking about their medication-taking behavior. This novel intervention will be used to empower patients with necessary information about their diseases, to address their beliefs around medication, to overcome barriers for nonadherence as well as to provide specific instruction to optimize the use of each medication on an individual basis [32, 33]. This technique emerged from previous alcohol and drug treatment literature, and the efficacy is supported by scientific evidence for the management of substance abuse and other chronic illnesses [34, 35]. This approach consists of the aforementioned four domains that will be used in this study: providing information about diseases, addressing beliefs on medication, overcoming barriers for nonadherence, and providing specific instructions to optimize the use of each medication. These domains are aligned to the MI principles and strategies described by Miller & Rollnick [19]. Before the initiation of the study, the researcher will receive training regarding MI techniques from a professional counselor. Training for MI skills consists of reading materials, viewing video demonstrations related to MI, and attending training with the counselor. Through this, the pharmacist will be equipped with essential skills to engage with the patients. The MI technique in this study will be carried out without a structured guideline to ensure the flow of the sessions, as studies have indicated the traditional method may produce poorer outcomes [36]. In addition, patients will be given knowledge alone is not sufficient regarding their medication, disease management, targeted clinical parameters and side effects of medication.

### Study procedure and follow up

**Fig 1:**
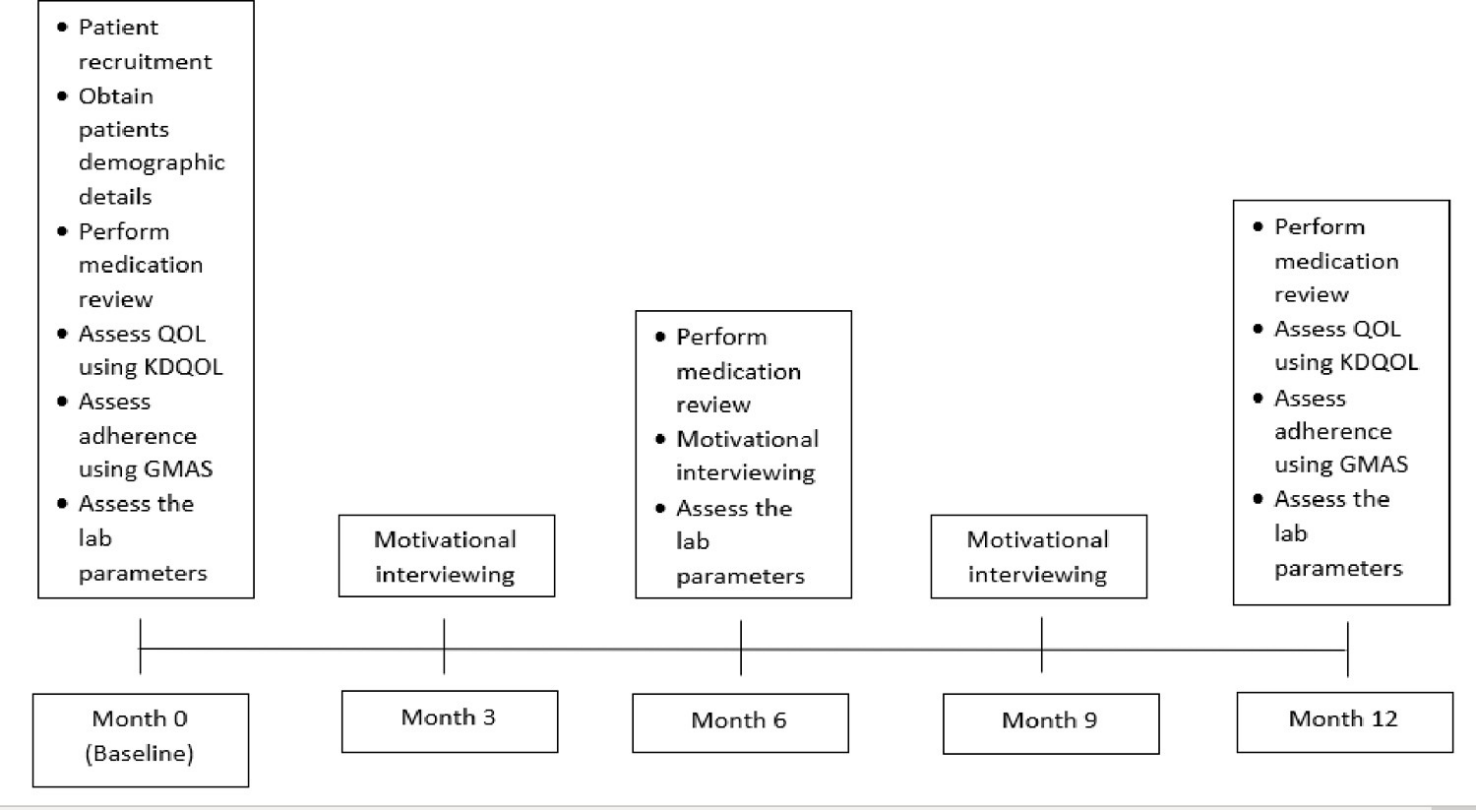
Flow chart summary of study procedure.

Once informed consent is obtained from the eligible patients at the first month’s visit, patients’ medication adherence, QOL and lab results—mainly the calcium, phosphate, hemoglobin, lipid profile, and glycosylated hemoglobin levels will be assessed. A medication review will also be performed. The updated list and observations/recommendations will be provided to the physician in charge. The medical records will be reviewed in the following six months to determine whether individual discrepancies have been corrected and whether MRP recommendations have been accepted by the prescriber. Motivational interviewing will be conducted in 3 sessions, in the 3^rd^, 6^th^ and 9^th^ months. Each session will last approximately 15 to 20 minutes for each patient. Only the first session will be done face to face (during the patient’s scheduled dialysis day) to build a good relationship with the patient, while the second and third sessions will be conducted over the phone to minimize close contact with the patients during the COVID-19 outbreak period. This method has proven its efficacy in previous research [37] and ensures safety for both patients and the researcher. The number of sessions to motivate and improve medication adherence has been set at three, as the optimal number is not known [38–40]. At the final visit (12 months), the medication review will be performed, medication adherence, QOL score and lab parameters will be assessed. The pre-post results will be checked for significance in improvement using the software package Statistical Package for Social Sciences (SPSS) Version 26.0. The results will be evaluated to check if there are any significant changes in the number of medication-related problems, adherence and QOL at the end of patient-centered pharmacist care. The summary of study procedure is shown in Fig 1.

All the recruited patients only can do their hemodialysis in the registered respective HD unit. They must strictly follow to their HD schedule, check blood sample every 3 months and reviewed by the nephrologist. This is the practice in the HD unit (at the study site) included. Patients’ movement control can be easily monitored, and the lab results will be captured in their medical records appropriately. Hence, missing data is avoided. As the interventions done every 3 months, the researcher can get the sufficient information from the patients and medical records. Patient missed appointments due to logistic reasons will be rescheduled within one week interval. However, missed appointment for more than 3 times due to hospitalization or changes in disease state, the case will be discussed with physician and co-researchers. Based on the expert opinion, decision on exclusion of the patient will be made.

#### Safety

Since this study is observational, there are no direct risks associated with participation.

#### Statistical analysis

Continuous variables will be expressed as mean ± standard deviation or median and interquartile range. Categorical variables will be expressed as a percentage. Adherence and QOL patterns before and after intervention will be assessed using paired sample t-tests or Wilcoxon signed-rank tests, depending on the sample distribution. Pearson correlation coefficient or Spearman rank correlation coefficient tests will be used to evaluate the associations between adherence and QOL. A general linear model will be applied to find the factors associated with MRPs, adherence and QOL. Data will be analyzed using SPSS Version 26.0. The significant level will be set at p < 0.05.

## Discussion

In Malaysia, the Ministry of Health has established the Renal Medication Therapy Adherence Clinic (MTAC), which is managed by pharmacists as one of the strategies in optimizing patients’ knowledge and implementing comprehensive strategies to increase adherence. However, the outcome and the impact of this Renal MTAC among hemodialysis are not known. In addition, the current Renal MTAC protocol only emphasizes patient education and medication counseling. A unique model with medication review and medication interviewing is needed to tackle the barriers around medication and increase patients’ belief in medication. Although MI is a promising tool, stronger evidence on its efficacy is needed to justify the use in the current Renal MTAC protocol. At the end of the study, through pharmacist roles in MR and MI, it is expected that the rate of medication problems will reduce, medication adherence and quality of life will improve, and clinical parameters will be optimized. If this is achieved, it will show pharmacists’ contribution to pharmaceutical care, and more hospitals will start indicating the need to have pharmacist-based pharmaceutical care.

### Study strengths & limitations

The limitation of this study is that it needs to be conducted as a pre-post study due to ethical consideration from the study site. Moreover, patients outcomes will be measured through questionnaire, recall bias and social-desirability bias may occur. Another limitation is the selection of 25% as the dropout rate for current study. As ESRD patients are high risk patients with high mortality and morbidity rates, an addition of 25% drop out rate to the total number of sample size may be an underestimation. Moreover, as the patient adherence will be measured using GMAS score, there is potential for overestimation of medication adherence and susceptibility to manipulation. However, patient clinical outcomes will be monitored to estimate an overall medication adherence rate. Nevertheless, medication adherence rate is susceptible to under or overestimation. The strength of this study is that it will be conducted in multicenter sites.

## Abbreviation

HD: hemodialysis
MRP: medication-related problems
MR: medication review
MI: motivational interviewing
QOL: quality of life
ESRD: end stage renal disease
GMAS: general medication adherence scale
KDQOL-36: Kidney Disease Quality of Life Short Form 36
LDL: low-density lipoprotein
CKD: chronic kidney disease
HRQOL: health-related quality of life
PCS: physical component summary
MCS: mental component summary
PCNE: Pharmaceutical Care Network Europe
MTAC: Medication Therapy Adherence Clinic
HKL: Hospital Kuala Lumpur
UMMC: University Malaya Medical Centre
